# The disruptive relationship among circadian rhythms, pain, and opioids

**DOI:** 10.3389/fnins.2023.1109480

**Published:** 2023-02-15

**Authors:** Jacob R. Bumgarner, Evan W. McCray, Randy J. Nelson

**Affiliations:** Department of Neuroscience, Rockefeller Neuroscience Institute, West Virginia University, Morgantown, WV, United States

**Keywords:** circadian rhythms, pain, opioids, opioid analgesia, circadian rhythm disruption

## Abstract

Pain behavior and the systems that mediate opioid analgesia and opioid reward processing display circadian rhythms. Moreover, the pain system and opioid processing systems, including the mesolimbic reward circuitry, reciprocally interact with the circadian system. Recent work has demonstrated the disruptive relationship among these three systems. Disruption of circadian rhythms can exacerbate pain behavior and modulate opioid processing, and pain and opioids can influence circadian rhythms. This review highlights evidence demonstrating the relationship among the circadian, pain, and opioid systems. Evidence of how disruption of one of these systems can lead to reciprocal disruptions of the other is then reviewed. Finally, we discuss the interconnected nature of these systems to emphasize the importance of their interactions in therapeutic contexts.

## 1. Introduction–Circadian rhythms

Pain, opioid analgesia, and opioid reward processing all are regulated by circadian clocks (Labrecque and Vanier, 1995; Bumgarner et al., 2021; Tamura et al., 2021). In turn, the pain and opioid processing systems, including the mesolimbic reward system, form a reciprocal relationship between one another and the circadian system. Moreover, the circadian, pain, and opioid systems are all influenced not only by circadian rhythms but by other rhythmic biological processes, including the sleep-wake cycle (Eacret et al., 2020; Palada et al., 2020). These systems form a complicated and poorly characterized temporal relationship with one another. Given that the US is currently at the peak of the opioid epidemic, the intertwined nature of these systems is important to consider in the context of pain management with opioid analgesic prescriptions (Ahmad et al., 2021).

Virtually all biological systems and processes display circadian rhythms. These rhythms are endogenous and cycle over a period of about 24 h in synchronization with the light-dark cycles of the environment (Reppert and Weaver, 2002). In mammals, circadian rhythms are present in processes ranging from cellular to systemic levels. Individual rhythms are often isolated and self-regulated, leading to a need for internal and environmental synchronization.

In mammals, circadian rhythms throughout the body are primarily synchronized by the suprachiasmatic nuclei (SCN) of the hypothalamus, commonly called the central pacemaker. To synchronize internal rhythms amongst one another and to the environment, the SCN integrates environmental photic signaling cues from the retina and relays this information in the form of neuronal and humoral signaling factors (Evans and Silver, 2015).

At the cellular level, 24-h rhythms are generated by a set of core clock genes that form an autoregulatory transcription-translation feedback loop. The intricate details of this loop have been described extensively elsewhere and extend beyond the scope of this review (Partch et al., 2014). Importantly, the proteins of the positive arm (CLOCK, BMAL1, and NPAS1) and negative arm of the feedback loop (PERs, CRYs), as well as ancillary loop proteins (e.g., REV-ERBα, RORα), regulate gene expression, and the activity of other signaling pathways. The regulatory action of the clock genes drives the rhythmic expression of approximately 40% of the rodent genome (Zhang et al., 2014). Further, up to 80% of the primate genome exhibits rhythmic expression in the context of other cyclical internal and external factors (Mure et al., 2018).

The regulatory dominance of the circadian system indicates the importance of its integrity for health. In the remainder of this review, we will examine how pain and opioid analgesia are regulated by circadian rhythms. We will then highlight how the disruption of the pain and mesolimbic reward systems can negatively impact the circadian system. We will conclude by highlighting the need to consider the relationship among these systems in clinical and therapeutic contexts.

## 2. Circadian rhythms of pain behavior and pain sensitivity

The function of the pain system is regulated by circadian clocks (Segal et al., 2018; Palada et al., 2020; Bumgarner et al., 2021). The precise relationship and nature of the circadian regulation of pain are still emerging, yet it is clear that circadian rhythms are present at all levels of organization within the pain system (Bumgarner et al., 2021; Mun et al., 2022). To further complicate the topic, pain rhythms are not consistent across species or even individuals, and other rhythmic processes such as sleep can also affect pain (Mun et al., 2022). Despite the still-emerging nature of the mechanistic drive behind circadian rhythms in pain, there is abundant clinical and preclinical behavioral evidence of this phenomenon.

Several rodent studies conducted between 1977 and 1994 began to characterize the nature of pain rhythms in mice. Two of these studies were the first to report the truly rhythmic nature of pain behavior. These two studies were conducted in constant conditions to reveal that rhythms of pain behavior were indeed circadian and not just driven by environmental effects. The first study observed behavioral pain rhythms in Syrian hamsters (*Mesocricetus auratus*) (Pickard, 1987), and the other observed rhythms in two strains of mice (*Mus*) (Oliverio et al., 1982).

Additional research during this period highlighted the complicated and inconsistent nature of the shape of these rhythms (Frederickson et al., 1977; Kavaliers and Hirst, 1983). Of these studies, one reported that rodent pain sensitivity peaked during the inactive phase (Kavaliers and Hirst, 1983), whereas others reported peaks during the active phase (Frederickson et al., 1977; Oliverio et al., 1982; Martínez-Gómez et al., 1994). Varying peak times have also been reported that were dependent on entrainment (Pickard, 1987). A further complication to this topic arose when one study reported antiphase (opposite) rhythms in C57BL/6 and Swiss Webster mice (Castellano et al., 1985). C57BL/6 mice were observed to have their shortest withdrawal latencies during the dark phase and the opposite occurred for the Swiss Webster mice (Castellano et al., 1985). It is possible that this discrepancy in behavior is a result of genetic differences between the two strains, as differences in underlying circadian biology between C57BL/6 and Swiss (CF1) mice have been previously noted (Pilz et al., 2015). A recent study examined time-of-day differences in mouse orofacial pain, reporting that peak behavioral pain responses were observed during the middle of the light (rest) phase (Niiro et al., 2021). The additional behavioral discrepancy between this study and the above studies may be a result of the different pain behavioral paradigms implemented. Each of these studies has provided important insight into the underlying biology of circadian rhythms in pain.

A major study was recently published to demonstrate the circadian nature of pain behavior in humans (Daguet et al., 2022), although only data on men were reported. The participants were held in a constant routine protocol that prevented photic environmental cues from entraining or masking behavior. Pain sensitivity was examined across the day along with other biological parameters. A circadian rhythm of pain sensitivity was observed, with pain sensitivity peaking in the middle of the night between 03:30 and 04:30 h. This rhythm was aligned with melatonin rhythms and antiphase to heart rate rhythms. Importantly, the authors were able to determine that the circadian system was the primary driving force of pain sensitivity rhythms. Homeostatic sleep pressure contributed to the rhythms, but only accounted for 20% of the rhythmicity (Daguet et al., 2022). The observed rhythmicity and rhythmic shape observed in this study are supported by previous models (Hagenauer et al., 2017) and other human studies examining pain threshold variations in humans outside of constant condition protocols (Zhu et al., 2022). In all, this seminal study provides additional evidence for the conserved nature of circadian rhythms of pain.

Rhythms of pain sensitivity are present in various states of disease and pain syndromes. However, pain rhythms are inconsistently altered in these states (Knezevic et al., 2022). For example, chronic pain conditions such as fibromyalgia and trigeminal neuralgia pain peak in the morning, whereas other neuropathic pain and temporomandibular joint pain peak in the night at 20:00 h (Knezevic et al., 2022). Variations in diurnal rhythms of acute pain also exist. For example, biliary colic and labor pain peak at night, whereas post-operative pain peaks in the morning (Knezevic et al., 2022). These variations may reflect differences in the timing of operations or underlying disease biology. Variations in peak pain intensity have also been observed in headache conditions (Burish et al., 2019). Other work examining a population of patients with heterogeneous chronic pain reported three differing active phase pain rhythms in separate clusters of patients (Tanaka et al., 2021). Finally, another study observed pain rhythms in patients with diabetic peripheral neuropathy or postherpetic neuralgia. Importantly, even when treated with gabapentin and nortriptyline, the rhythms were blunted, yet persisted (Gilron et al., 2013).

The mechanisms behind behavioral pain rhythms are complex and not well-understood. There is growing evidence of the clock gene-driven rhythmic expression of various proteins involved in nociception and pain signal processing (Chu et al., 2022). For example, rhythmic expression of pain-related proteins in mice observations include substance P (Zhang et al., 2012) and the α2δ-1 voltage-gated calcium channel in the dorsal root ganglia (Kusunose et al., 2010), *Trpa1* in the trigeminal ganglia (Niiro et al., 2021), and the NR2B NDMA glutamate receptor subunit and two of its response elements in the spinal cord (Xia et al., 2016). Lastly, and of critical relevance, rhythmic expression of the μ-opioid receptor transcript has been observed in the periaqueductal gray of mice (Takada et al., 2013). Additional insight into the potential molecular, cellular, or network-driven mechanisms behind circadian rhythms and pain have been extensively characterized elsewhere (Segal et al., 2018; Bumgarner et al., 2021). Moreover, the mechanisms behind these behaviors become particularly complicated when considering the heterogenous phenotypes of pain in the context of disease or neuropathy. Early insight into this complicated topic has also been provided elsewhere (Warfield et al., 2021).

Taken together, abundant behavioral and emerging mechanistic evidence highlights the nature of circadian rhythms of pain behavior. The weight of this evidence becomes particularly important when considering and developing strategies to optimize pain therapeutics. The timing of therapeutic interventions for pain can and should continue to be optimized. This is particularly true in the context of pharmacological treatments, including opioids.

## 3. Circadian rhythms of opioid analgesia

Just as pain behavior varies across the day, the efficacy of opioid analgesics also varies across the day (Labrecque and Vanier, 1995). Opioid analgesic requests and self-administration also vary across the day. The functional relationship between the circadian and reward/opioid systems continues to be well-characterized and is therefore less elusive than the nature of circadian rhythms of pain (Becker-Krail et al., 2022b). However, the exact underlying mechanisms that drive daily variations in opioid analgesic efficacy are still not entirely understood. These variations are likely dependent on both circadian rhythms and sleep. Numerous clinical and preclinical studies have shed light on the underlying biology of the relationship between circadian rhythms, pain, and opioid efficacy.

First, opioid analgesic efficacy across the day has been examined in healthy human participants. One study examined the effect of fentanyl analgesia on women and men participants at four time points across the day (Boom et al., 2010). The peak analgesic effect occurred at 17:30 h, and the trough occurred at 5:30 h. Interestingly, fentanyl-induced hyperalgesia occurred around 2 h after the dosing, but this effect only occurred at the 2:00 h time point (Boom et al., 2010). This effect might have important clinical implications.

Several studies have examined diurnal variations in postoperative opioid analgesia. In one study, participants were administered morphine either subcutaneously every 4 h or *via* a continuous intravenous infusion for 3 days following thoracic or abdominal surgery (Labrecque et al., 1988). Rhythmic variations in pain reports were observed, but only on the first day of the study. The peak of pain reporting was around 1900 h for the subcutaneous group and around 18:30 h for the IV group (Labrecque et al., 1988). Importantly, as there were no saline control groups, we are unable to infer whether the morphine administration drove rhythms in pain intensity or if they just altered the rhythms. Moreover, there was no report on the timespan between the conclusion of surgery and the onset of access to self-administration (Labrecque et al., 1988). Another study examined time-of-day differences in morphine self-administration in patients following gastric bypass surgery (Graves et al., 1983). Doses were recorded every 2 h for 3 days. The peak of analgesic usage was observed at 09:00 h, and the trough occurred at 03:00 h (Graves et al., 1983). A third study examined hospital-wide patient-controlled opioid delivery following a variety of surgical operations (Sandoval, 2018). Consumption was recorded in four blocks; the block between 23:00 and 05:00 h was observed to have the lowest incidence of self-administration (Sandoval, 2018). The self-administration nature of these studies highlights a relationship between daily rhythms in pain and opioid analgesia. These results also highlight related time-of-day variations in reward processing, although this is beyond the scope of the current review.

Diurnal variation in opioid efficacy has also been characterized in patients with chronic pain conditions. One study examined the morphine self-administration rates in post-operative cancer patients (Auvil-Novak et al., 1990). In this study, the delivery of morphine peaked between 08:00 h and 12:00 h, and significant 12- and 24-h patterns of self-administration were observed (Auvil-Novak et al., 1990). In a second study, pain and morphine consumption were measured in 4-h blocks for 2 days after surgery (Auvil-Novak et al., 1990; Boscariol et al., 2007). Time-of-day differences in pain symptoms were observed, with peak pain scores occurring during the 8:00–12:00 h block. Further, on the second postoperative day, there was a time-of-day difference in morphine consumption, with greater consumption occurring from 4:00 to 12:00 h, although this effect was not present on the first postoperative day. Importantly, the temporal variations in pain persisted even in the presence of opioids (Auvil-Novak et al., 1990; Boscariol et al., 2007).

Opioid efficacy across the day has also been examined in patients with varying neuropathic pain conditions. One study examined the effect of time of day on pain intensity ratings in patients with diabetic neuropathy (Odrcich et al., 2006). Indeed, there were diurnal variations in pain intensity reporting; pain intensity increased as the day progressed. Pain rhythmicity was also assessed in the context of morphine, gabapentin, or combination treatment. Unsurprisingly, analgesic treatment reduced pain intensity across the day, but the rhythms of pain across the day persisted (Odrcich et al., 2006). Of note, this study did not differentiate between the contributions of homeostatic sleep pressure versus circadian rhythms to the rhythmic pain ratings. Three additional studies examined time-of-day variations in opioid administration in patients with cancer pain. One of these studies reported peak morphine doses in cancer patients during the night (Citron et al., 1992). In contrast, the other two studies reported greater requests for opioids during the day and evening (Bruera et al., 1992; Gagnon et al., 2001). Differences among these studies may be a result of one or multiple combined factors, including differences in opioids used or the type of cancer present. Differences also may be a result of unrecorded differences in sleep-wake cycle disturbance or circadian rhythm disruptions during the hospital stays.

Preclinical examinations of the time-of-day effects of opioid analgesia have provided some insight into the potential mechanisms in the variations of opioid analgesic efficacy. Importantly, there are circadian rhythms in the function of pain neurocircuitry that might modify analgesic efficacy (Bumgarner et al., 2021) as well as mesolimbic reward circuitry that might modify self-administration rates (Becker-Krail et al., 2022b). As mentioned above, one study reported diurnal differences in the expression of μ-opioid receptor transcript in the periaqueductal gray of control mice and mice with partial sciatic nerve ligations (Takada et al., 2013). Another study observed diurnal variations in endogenous leu- and met-enkephalin in both the hypothalamus and hippocampus of male rats, an effect noted to have potential implications for exogenous morphine processing (Miguel Asai et al., 2007). Opioid receptor binding also varies across the day in male rats. To study this, naloxone was administered to the rats and brain concentrations were recorded following tissue collection (Naber et al., 1981). The peak concentration of naloxone was recorded during the dark phase at 22:00 h. This effect likely reflects diurnal differences in receptor availability, turnover, or xenobiotic metabolism (Naber et al., 1981).

Other preclinical studies have examined diurnal variations in morphine analgesia. Two studies examined morphine analgesia in healthy mice and reported greater analgesic peaks toward the beginning of the active phase (Morris and Lutsch, 1967; Frederickson et al., 1977). Persistent rhythms of pain responsiveness were observed in these studies even in the presence of opioids, an effect that is consistent with human observations. An additional study on the role of nitric oxide synthase on diurnal opioid analgesic efficacy provided further insight into a mechanism that might drive this effect (Güney et al., 1998). Morphine administration alone had diurnal analgesic effects, but inhibition of nitric oxide synthase potentiated morphine analgesia maximally during the dark phase. These results implicate nitric oxide in morphine analgesic modulation and suggest that there is a time-of-day relationship between opioid-induced analgesia and other related signaling pathways.

Considered together, there are clear diurnal variations in the efficacy of opioid analgesia, the rates of self-administration of opioids in clinical contexts, and the underlying biological systems upon which opioids act to induce analgesia. These diurnal effects have obvious clinical implications, but also indicate the need to consider patient education in the context of opioid analgesic prescriptions. Future work may seek to alter the individual timing of opioid administration to optimize the analgesic efficacy. As noted, the integrity of the circadian system is critical for health and optimal functioning. The consideration of the importance of circadian rhythms in pain and opioid analgesia leads to an important question, *viz*., can pain and opioids disrupt the circadian system?

## 4. Circadian rhythm disruption, pain, and opioids: A feedback loop

There is a feedback loop formed by the circadian and pain systems, as well as the systems that are modulated by opioids, including the mesolimbic reward circuitry. Disruption of one of these systems can lead to negative alterations in the others (Webb, 2017; Tamura et al., 2021; Becker-Krail et al., 2022b; Mun et al., 2022). This disruptive feedback connected to the circadian system has important consequences in the context of opioid analgesia and substance misuse (Hasler et al., 2012; Logan et al., 2018; Barko et al., 2019; Hasler and McClung, 2021). Over the next two subsections, we will discuss evidence demonstrating the disruptive relationship among these systems and highlight the potential clinical consequences of the interaction among these systems.

### 4.1. Disruption of circadian rhythms and pain

Disruption of circadian rhythms is a pervasive and yet nearly ubiquitous aspect of modern human life (Bumgarner and Nelson, 2021). Circadian rhythm disruption can occur *via* environmental disruptors, such as light at night, or can occur *via* behavioral disturbances, such as jet lag, social jet lag, or mistimed eating (Vetter, 2020). Disruption of circadian rhythms can affect individual or several biological rhythms, and in general, these disruptions are directly or indirectly associated with several negative health consequences (Walker et al., 2020). Notably, disrupted circadian rhythms can negatively affect pain. These effects have been observed in both clinical and preclinical contexts.

Most of the evidence demonstrating the negative effects of circadian rhythm disruption on human pain responses comes from night shift work studies. Generally stated, night shift work exacerbates pain perception. One night of shift work was observed to increase cold pain perception (Pieh et al., 2018). Another study concluded that night shift work was associated with increased pain score ratings in response to noxious heat and electric stimuli, but not too cold stimuli (Matre et al., 2017).

Several longitudinal studies of nurse night shift workers demonstrate that night shift work increases the risk for lower back pain (Zhao et al., 2012; Takahashi et al., 2015), with larger increased risk ratios for obese night shift working nurses (Zhao et al., 2012). An additional study reported that night shift work in nurses was associated with increased sick leave for lower back pain-related issues, among other issues (Eriksen et al., 2004).

Sleep disruption and exacerbated pain also form a reciprocal relationship. However, this relationship is not entirely distinct from circadian rhythm disruption. For example, a meta-analysis of five studies reported that sleep deprivation leads to an increased perception of pain (Schrimpf et al., 2015). Another study examined the relationship among circadian activity, sleep quality, and pain in cancer patients (Ma et al., 2014). A correlative and disruptive relationship was observed. Twenty-four autocorrelation coefficients of activity were predictive of pain intensity; pain intensity was correlated with sleep quality, and greater pain was associated with less regular rest/activity rhythms (Ma et al., 2014). The precise causative relationship of each variable assayed in this study is difficult to pinpoint, but this is likely because each variable contributes to the overall disruptive health effects. Lastly, a longitudinal study examined the relationship among sleep quality, chronic musculoskeletal pain, and widespread pain (Skarpsno et al., 2021). Long-term poor sleep quality and a transition from good to poor sleep quality during the study were associated with increased risk ratios for chronic pain. However, even individuals that transitioned from poor to good sleep quality during the study had increased risk ratios for chronic pain (Skarpsno et al., 2021). Taken together, these studies indicate that sleep pressure alone can heighten pain sensitivity and that sleep disruption, circadian rhythm disruption, and pain form a disruptive relationship.

Preclinical studies have also examined the effects of circadian rhythm disruption on pain. One study examined how molecular alterations to the clock gene feedback loop altered fentanyl-induced hyperalgesia in NPAS2-deficient female and male mice (Puig et al., 2022). A striking sex difference was observed; only male NPAS2-deficient mice exhibited fentanyl-induced hyperalgesia. This study is amongst the first to provide mechanistic insight into how disrupted circadian rhythms can impact pain behavior (Puig et al., 2022). A second preclinical study determined that exposure to dim levels of artificial light at night (5 lux) exacerbated sensitivity to noxious cold stimuli and reduced mechanical withdrawal thresholds in male mice (Bumgarner et al., 2020). Another study similarly reported alterations in mechanical withdrawal thresholds in a simulated jet lag paradigm (Das et al., 2018). A final study examined the effect of mistimed eating on mechanical withdrawal thresholds in a mouse model of neuropathic pain (Xu et al., 2018). Food consumption restricted to the inactive phase exaggerated mechanical allodynia in male mice with chronic constriction injury (Xu et al., 2018).

Finally, correlative evidence indicates that pain may disrupt circadian rhythms. For example, conditions such as cancer (discussed above) and fibromyalgia are associated with altered activity and circadian rhythms (Korszun, 2000; Savvidis and Koutsilieris, 2012). However, the heterogenous nature of these diseases makes it difficult to isolate the exact contribution of pain to the observed disrupted rhythms. Further, non-human animal studies of spinal cord injury and partial sciatic nerve ligation have also reported disrupted molecular rhythms (Odo et al., 2014; Gaudet et al., 2018). Both models are frequently used to examine neuropathic pain, but without explicit examination of the role of pain in these studies, one can only infer the degree of connection between pain and the observed altered rhythms. Further examination of the potential for pain to disrupt circadian rhythms is warranted.

Taken together, it is apparent that disrupted circadian rhythms negatively affect pain, and additional correlative indicates that pain might also alter circadian rhythms. Continued research is needed to understand the mechanisms that drive this relationship. This approach could improve future therapeutic approaches by identifying novel therapeutic targets or could drive non-pharmacological and non-invasive treatment strategies for pain management, such as wearing blue light-filtering glasses during the night.

### 4.2. Disrupted circadian rhythms and opioids

Opioids can disrupt the integrity and timing of the circadian system (Tamura et al., 2021). This is true both in the context of opioid administration for analgesic purposes and disordered opioid use. Further, disruption of the integrity of the circadian system can alter the efficacy and effects of opioids in therapeutic and substance misuse contexts (Becker-Krail et al., 2022b).

Much of the evidence exploring the mechanisms behind these effects comes from studies examining animals with genetic manipulations to the circadian clock, as this approach can provide strong insight into the role of these genes on behavior (Nelson and Young, 1998). For example, one study assessed the role of *Per2* on morphine analgesic tolerance in the context of noxious heat stimulation by studying *Per2*^*Brdm*1^ mutant mice (Perreau-Lenz et al., 2010). *Per2*^*Brdm*1^ mice carry a mutation in the PAS domain of the *mPer2* gene; mice with this mutation exhibit diminished rhythmic expression of *mPer2* as well as *mPer1* (Zheng et al., 1999). Morphine reduced heat sensitivity in both the wild-type control mice and the *Per2* mutant mice, but *Per2* mutations led to reduced morphine tolerance over several days of injections, thereby increasing the analgesic efficacy of the drug relative to the wild types as the injections progressed. Further, *Per2* mutant mice in this study exhibited reduced global withdrawal scores following naloxone-precipitated withdrawal at the end of the injection series (Perreau-Lenz et al., 2010). Another study determined that astrocytes in the nucleus accumbens display rhythmic transcriptomic and metabolic profiles across the day (Becker-Krail et al., 2022a). Disruption of these rhythms altered reward-related behaviors and excitatory neurotransmission in the nucleus accumbens during the light phase, highlighting the importance of intact mesolimbic glial circadian rhythms for reward processing (Becker-Krail et al., 2022a). Lastly, the role of *Per1* on morphine-conditioned place preference was assessed by administering a DNAzyme that cleaves the *mPer1* transcript (Liu et al., 2005). *Per1* transcript knockdown altered the acquisition phase of the conditioned place preference test, but it did not alter the expression phase of the test in mice (Liu et al., 2005). Together, these studies implicate the importance of the integrity of the circadian system for opioid processing both in the context of analgesia and reward processing. However, from these results, it was not necessarily apparent whether disruption of circadian rhythms was necessarily detrimental in the context of analgesia.

Opioid administration can also directly influence the circadian system and behavioral rhythms, including sleep. Numerous clinical and preclinical studies support this claim. For example, fentanyl administration can phase-shift circadian rhythms in hamsters (Vansteensel et al., 2005). This effect is dependent on opioid receptor binding, as phase shifts were abolished in the presence of naloxone (Vansteensel et al., 2005). Moreover, fentanyl altered the effects of dark-phase light pulses on locomotor activity and *Per1* and *Per2* expression (Vansteensel et al., 2005). Acute (one injection) and chronic (2 injections/day for 7 days) fentanyl treatment can also alter sleep in mice by reducing sleep bouts and the duration of non-rapid eye movement (NREM) sleep (Gamble et al., 2022). Deficiency of NPAS2, a core clock gene, exaggerates these effects (Gamble et al., 2022).

Morphine administration can also modulate circadian rhythms. For example, administration altered the peak of locomotor rhythms and the phase of activity in male rats (Glaser et al., 2012). The direction of these effects was inconsistent throughout the study, but the disruptive aspect of morphine on behavioral rhythms was consistent (Glaser et al., 2012). Administration of a delta opioid receptor agonist, BW373U86, led to phase advances in free-running Syrian hamsters, indicating the direct effects of opioid receptor stimulation on circadian synchronization (Byku and Gannon, 2000). In contrast, agonism and antagonism of the mu, kappa, and delta opioid receptors did not affect free-running rhythms of mice in this study (Byku and Gannon, 2000). Morphine administration can differentially alter basal neural activity in the paraventricular nucleus of the thalamus (McDevitt and Graziane, 2019). Light-phase morphine administration increased spontaneous neural activity in this region, whereas dark-phase administration did not (McDevitt and Graziane, 2019). Lastly, morphine withdrawal in rats disrupts clock gene rhythms in various cerebral loci and peripheral blood mononuclear cells (Li et al., 2009b).

Additional evidence indicates that opioids can also modulate circadian rhythms in humans. One large-scale post-mortem study examined the effects of opioid use disorder on rhythmic transcriptomic profiles in humans (Xue et al., 2022). By using the time of death as a time-of-day marker, an around-the-clock tissue bank was created from the post-mortem tissue collections. Rhythmic transcripts were observed in the nucleus accumbens and the ventral tegmental area of unaffected individuals and individuals with opioid use disorder, but there was minimal group overlap in the rhythmic transcripts. Interestingly, in individuals diagnosed with opioid use disorder, the nucleus accumbens had greater transcriptomic rhythmicity and the ventral tegmental area displayed reduced transcriptomic rhythmicity than the unaffected individuals (Xue et al., 2022). Importantly, the alterations in the transcriptomic profiles of these individuals were associated with opioid, dopamine, and GABA neurotransmission, as well as altered synaptic function (Xue et al., 2022). Another study examined the effect of heroin withdrawal on clock gene rhythms in human patients. Thirty days of withdrawal were associated with altered clock gene rhythms in peripheral blood mononuclear cells (Li et al., 2009a). Serum concentrations of various hormones and cytokines were also disrupted by heroin withdrawal (Li et al., 2009a).

Taken together, the evidence presented in this section highlights the disruptive interaction between the circadian system and opioids. Preclinical studies have provided mechanistic insight into these effects, with strong evidence pointing to the role of clock genes on both opioid-mediated disruption of the circadian system, as well as clock gene signaling integrity on opioid processing. The disruptive effects of opioids include altered synchronization and integrity of circadian rhythms in clock gene expression, neural activity, and behavior. In isolation, these effects may be considered manageable, but out of isolation, they can create a spiraling feedforward loop between the pain and circadian systems.

## 5. Conclusion and future directions

The circadian system, pain system, and opioid processing pathways form a spiraling and disruptive feedback loop. Opioids can disrupt circadian rhythms, circadian rhythms modulate opioid analgesia and opioid processing, and disrupted circadian rhythms can alter the efficacy of opioids. In turn, circadian rhythms regulate pain behavior and sensitivity across the day, and circadian rhythm disruption can exacerbate pain behavior. This loop is closed *via* the effects of opioids on pain and pain hypersensitivity. In basal contexts, the relationship among circadian rhythms, pain, and opioid processing is likely not of significant importance, but the components of this relationship are particularly important to consider in the context of disease and disordered substance use.

Given the dire state of the opioid epidemic in the United States, additional understanding and consideration of this loop will likely improve therapeutic interventions for pain. Moreover, the interaction among the circadian, pain, and opioid processing systems highlights the need for the development and implementation of non-pharmacological interventions. These interventions may seek to mitigate circadian rhythm disruption in pain patients, or they might target the circadian system to manage pain, such as recent and promising light therapies (Leichtfried et al., 2014; Ibrahim et al., 2017; Martin L. et al., 2021; Martin L. F. et al., 2021; Martin et al., 2022). Pain also has to potential to disrupt sleep at night, which in turn can lead to a spiraling disruption of other rhythms. As discussed above, these effects can lead to increased opiate use. For this reason, sleep medicine should be equally considered in the context of alternative and supplementary pain therapeutic interventions. In conclusion, circadian rhythms, pain, and opioids form a complex, yet demonstrably interconnected relationship. This relationship likely diminishes therapeutic outcomes when not considered and may even be contributing to the ongoing opioid epidemic.

## Author contributions

JB and RN conceived the idea behind the manuscript. JB and EM conducted the research for the manuscript. All authors wrote and edited the manuscript and approved the submitted version.
